# The effect of older age and stage on risk of recurrence and mortality following curative resection, in a national cohort of patients with colorectal cancer

**DOI:** 10.1038/s41416-026-03553-4

**Published:** 2026-07-21

**Authors:** Orouba Almilaji, Kate Walker, Jan Van Der Meulen, Nicola Fearnhead, Michael Braun, Linda D. Sharples

**Affiliations:** 1 Department of Health Service Research and Policy, Faculty of Public Health and Policy, London School of Hygiene and Tropical Medicine, London, UK; 2 Clinical Effectiveness Unit, The Royal College of Surgeons of England, London, UK; 3 Department of Colorectal Surgery, Cambridge University Hospitals NHS Foundation Trust, Hills Road, Cambridge, UK; 4 Department of Oncology, The Christie NHS Foundation Trust, Manchester, UK; 5 Department of Medical Statistics, Faculty of Epidemiology and Public Health, London School of Hygiene and Tropical Medicine, London, London, UK

**Keywords:** Oncology, Health care

## Abstract

**Background:**

Older patients are routinely excluded from clinical research, limiting understanding of recurrence and mortality in colorectal cancer (CRC) patients over 75 who undergo curative major resection.

**Methods:**

Using linked data from national cancer, treatment and administrative datasets, we estimated recurrence, CRC-related and non-CRC mortality to 5 years after surgery for patients selected for curative resection in England between 2014 and 2020, who survived >9months. Recurrence was defined using diagnostic and treatment codes.

**Results:**

50,480 patients had curative resection, 15,496 (30.7%) aged >75. Recurrence-free survival at 5-years after resection was 66.2% (95%CI: 65.4%, 67.0%) for patients aged >75 and 77.4% (77.0%, 77.9%) for patients aged 18-75. By 5-years, 13.3% (12.7%, 13.9%) of the older group had died from CRC and 14.2% (13.6%, 14.8%) from non-CRC causes, compared with 7.7% (7.4%, 8.0%) and 3.6% (3.4%, 3.8%) respectively in the younger group. Recurrence-free survival was 22% lower for cancer stage 3 versus stage 1, and 12–13% lower for patients aged >75 versus ≤75.

**Conclusions:**

Cancer stage was the strongest predictor of recurrence and CRC-related death. In patients selected for surgery, those aged>75 had similar recurrence risk to younger patients, but higher risk of death following recurrence, and were as likely to die from other causes as from CRC.

## Introduction

Colorectal cancer (CRC) is the third most common cancer and the second most common cause of cancer death in the UK, accounting for 10% of all cancer deaths (2017–2019) [1]. If feasible, surgery offers the best chance of long-term survival, with 2-year survival after any curative or palliative resection in England and Wales increasing to 85% in 2019/2020 [2]. A major objective of longer term management after curative resection is the early detection and treatment of recurrence or progression of disease, which has been estimated to occur in 20–30% in a Danish population study of CRC patients aged under 80 years [3].

Between 2014 and 2019, approximately 30% of CRC patients who had curative resection in the UK were aged over 75 years at diagnosis. ([4], Table 1). Despite this, CRC patients aged over 75 are often excluded from randomised trials and other research, so that their outcomes are less well studied [5]. Older adults who do participate in cancer trials tend to be highly selected, with fewer comorbidities and functional limitations than patients of a similar age treated in clinical practice [6]. Most older patients have more comorbidities and different tumour characteristics to younger patients. Therefore, it is important to understand the timing of recurrence in relation to competing risks, such as death from other causes. Robust estimates of recurrence incidence and prevalence over time, and their relationship to death from CRC and non-CRC causes, are urgently needed to support informed decision-making and patient counselling in older patients.

**Table 1:** Patient and tumour characteristics at the time of diagnosis of colorectal cancer for all patients who underwent curative resection and for patients who had recurrence at any time during follow up.

Variable at baseline	Level	All patients (N = 50,480)	Patients who had recurrence during follow up (N = 9,517)
Age category	18–60	11,364 (22.5%)	2,267 (23.8%)
	61–65	6,011 (11.9%)	1,059 (11.1%)
	66–70	8,885 (17.6%)	1,617 (17.0%)
	71–75	8,724 (17.3%)	1,613 (17.0%)
	76–80	7,667 (15.2%)	1,514 (15.9%)
	81–98	7,829 (15.5%)	1,447 (15.2%)
Age (years)	Mean (SD)	68.8 (11.4)	68.8 (11.7)
Sex	Male	27,949 (55.4%)	5,603 (58.9%)
	Female	22,531 (44.6%)	3,914 (41.1%)
Ethnicity	Black	683 (1.4%)	148 (1.6%)
	South Asian	1,140 (2.4%)	211 (2.3%)
	White	45,477 (94.4%)	8,747 (94.3%)
	Mixed	161 (0.3%)	30 (0.3%)
	Other	720 (1.5%)	139 (1.5%)
	Missing	2,299	242
Index of Multiple Deprivation	Least deprived 1	11,994 (23.8%)	2,223 (23.4%)
	2	11,795 (23.4%)	2,192 (23.0%)
	3	10,460 (21.1%)	1,929 (20.3%)
	4	8,809 (17.5%)	1,718 (18.1%)
	Most deprived 5	7,242 (14.4%)	1,455 (15.3%)
Charlson index	0	34,583 (68.5%)	6,296 (66.2%)
	1	11,334 (22.5%)	2,245 (23.6%)
	2	3,346 (6.6%)	713 (7.5%)
	>2	1,217 (2.4%)	263 (2.8%)
Histological Grade of cancer	1	1,843 (3.8%)	268 (3.0%)
	2	40,686 (84.6%)	7,443 (83.0%)
	3–4	5,553 (11.6%)	1,253 (14.0%)
	Missing	2,398	553
Overall cancer stage	1	13,963 (27.7%)	1,393 (14.7%)
	2	22,221 (44.1%)	3,693 (38.9%)
	3	14,164 (28.1%)	4,410 (46.4%)
	Missing	132	21
T stage	T1	5,537 (11.0%)	506 (5.4%)
	T2	10,307 (20.4%)	1,284 (13.6%)
	T3	27,554 (54.9%)	5,297 (56.1%)
	T4	6,751 (13.5%)	2,355 (24.9%)
	Missing	331	75
N stage	N0	36,106 (72.0%)	5,073 (53.7%)
	N1	9,680 (19.3%)	2,690 (28.5%)
	N2	4,349 (8.7%)	1,685 (17.8%)
	Missing	345	69
Site of tumour	Colon	35,506 (70.3%)	6,028 (63.3%)
	Rectum	14,974 (29.7%)	3,489 (36.7%)

National electronic health records (EHR) are increasingly used for audit, clinical epidemiology and long-term follow up in randomised clinical trials (RCTs) [7]. EHR offer an inclusive approach to data capture, enabling analysis of patient groups often excluded from RCTs, such as older patients and those with multiple comorbidities, so that results are more widely applicable. They are particularly useful for long-term outcomes, but recurrence is not accurately recorded in national EHR. A clinical rule-based algorithm for incidence and timing of CRC recurrence using 279 four-digits ICD-10 diagnosis and OPCS-4 treatment codes, and 3 administrative codes has been developed and validated, allowing recurrence and recurrence-free survival to be estimated in a large representative population [4].

The aim of this paper is to report recurrence and survival following curative resection, in an unselected national cohort of patients undergoing curative resection for CRC, with respect to age and overall cancer stage [8]. We use multistate models to allow for the shared risk factors for different events (recurrence, CRC death and non-CRC death) and appropriately account for censored follow-up times due to these inter-dependent competing events.

## Methods

### Data sources

The study used five linked national datasets containing secondary care episodes provided by UK National Health Service (NHS) hospitals in England. Datasets were the Hospital Episode Statistics Admitted Patient Care (HES APC) and Outpatients (HES OP) datasets [9], the National Cancer Registry (CR), the Radiotherapy (RTDS) dataset and the Systemic Anticancer Therapy (SACT) dataset [8, 10, 11]. Patients are recorded prospectively in these administrative and disease-specific databases at the time of presentation to secondary care. Datasets were supplied as pseudonymised data, linked at patient level. See Supplement for further details of data sources for each data item.

### Participants

The analysis cohort included all CRC patients without metastatic disease aged ≥18 years at diagnosis, who had a major resection with curative intent between July 2014 and September 2020. Patients were considered to have had a major resection with curative intent, which we refer to as a “curative major resection”, if they underwent an elective major resection in the period 31 days before to 1 year after CRC diagnosis in HES-APC or CR datasets, and who were alive with no evidence of recurrence in the data 9 months after curative treatment (See Supplement Figure SF1 for study population flow chart). Metastases were defined as stage M1, overall cancer stage 4 or Dukes’ D or missing data in all three of these variables in the CR [12].

To ensure that evidence of recurrence in the data was most likely to be related to CRC, we excluded patients who had another primary cancer recorded in CR or HES-APC in the 12 months before CRC diagnosis to end of follow up, or who had diagnosis of a secondary malignant neoplasm 12 months before CRC diagnosis to 6 months after in HES-APC or CR.

### Recurrence definition

Evidence of recurrence, based on ICD-10 diagnosis and OPCS-4 procedure codes [13, 14], was extracted from all five datasets. We defined recurrence as the earliest recorded diagnosis or treatment of recurrence (diagnoses of recurrence, procedures for local/regional progression, procedures for metastatic disease, chemotherapy, radiotherapy, or palliative care) between 9 months and 5 years (the recurrence window) after curative major resection for the primary CRC, according to the clinical rule-based algorithm developed by Almilaji et al. [4] (See Supplement Table ST1 for ICD-10 and OPCS-4 codes). To be consistent with Almilaji’s definition [4], we assumed that any cancer-specific treatments observed in the 9 months immediately after curative resection were either adjuvant therapies or for treatment of persistent disease.

### Outcomes

**Fig. 1:**
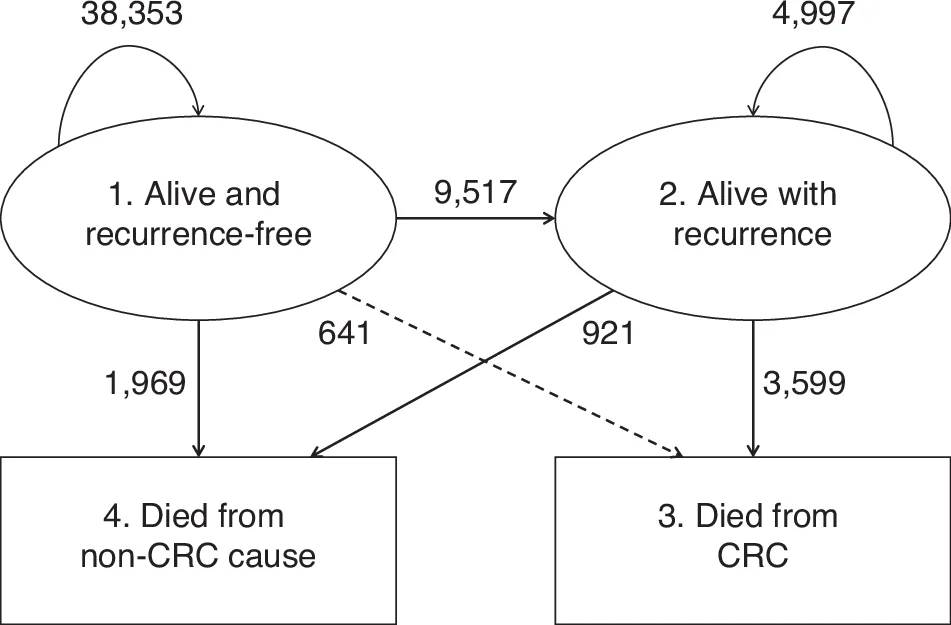
Multistate model showing possible transitions between states. Annotated figures are the numbers of observed transitions between states during the recurrence window (9 months to 5 years after resection) and the number of people who were alive and recurrence-free (n = 38,353) or alive with recurrence (n = 4,997) at 5 years after resection.

We defined CRC and non-CRC death using underlying cause of death ICD-10 codes from the Office of National Statistics mortality data, provided in the HES APC dataset (see Supplement for specific cause of death codes). During follow up, we considered four possible health states: (1) alive and recurrence-free; (2) alive with recurrence, (3) died from CRC and (4) died from non-CRC causes (Fig. 1). All deaths within 9 months of resection were assumed to be related to the primary CRC or complications of surgical resection, and were excluded. All remaining patients were assumed to start follow up in state 1, alive and recurrence-free, at 9 months after curative major resection for CRC (analysis time zero). Thereafter, patient pathways progress according to the transitions between health states. Some patients died from CRC with no evidence of recurrence in the data (dashed line in Fig. 1). This observed direct transition from recurrence-free to CRC death, without first having recurrence, is unlikely to represent the underlying disease process but allows us to investigate factors associated with non-capture of recurrence in the data prior to CRC death. Follow up time ends either at death, 5 years after the curative major resection, or 30/6/2022, whichever is earlier.

### Descriptive analysis

After constructing the dataset, we summarised patient and tumour characteristics recorded at CRC diagnosis. We considered patient characteristics (age at diagnosis, sex, Index of Multiple Deprivation (IMD), ethnicity and Charlson index), and tumour characteristics (histological grade, overall cancer stage, tumour stage, nodal involvement and whether the primary tumour was in the colon or rectum). For cancer staging we extracted codes labelled “Stage_best”, “T_best” and “N_best” from the National Cancer Registry, which use both clinical and pathological stage in their definition [8]. These are described as overall cancer stage, T stage and N stage throughout. Relationships between age groups and other ordered categorical variables were assessed by Kendall’s τ (type C) correlation coefficient for categorical variables. Values of τ that were less than 0.10 are described as very weak correlation and values between 0.11 and 0.20 as weak correlation [15]. We used separate Kaplan-Meier plots to explore time to recurrence, time to CRC death and time to non-CRC death and time after recurrence to each cause of death (Fig. 1). We explored Cox proportional hazards models for each transition separately to assess potential impact of patient and tumour characteristics. In these exploratory analyses, all other outcomes were censored.

### Design and construction of multistate model (MSM)

Times to recurrence of disease, CRC death and death from other causes are not independent, therefore separate estimation of each outcome produces biased estimates [16]. To address this, we used a multistate model that simultaneously estimates all five possible transitions between states: three transitions from the initial state of ‘alive and recurrence-free’; and two transitions from recurrence to either CRC death or death from other causes (Fig. 1) [17]. (see Supplement for details) This approach handles death as a competing event, thereby avoiding bias in estimating incidence of events [16], and allows multiple events in the same patient to be included, thereby improving accuracy of estimates. The model assumes that covariate effects on hazards are proportional for all transitions. Whilst this is unlikely to hold, it allows estimation of “average” hazard ratios for each transition over follow up using a Cox model. We used the Aalen-Johansen survival method to estimate cumulative incidence rates and the proportion in each state during the recurrence window [16]. Models were fitted using restricted maximum likelihood with robust standard errors [18]. All analysis used Rstudio version 2024.04.2.

### Sensitivity analyses

Because direct transition from recurrence-free to CRC death, without first having recurrence, is unlikely to represent the underlying disease process, we carried out sensitivity analyses in which we (i) refitted the models excluding these patients and (ii) fitted a model in which patients who died from CRC were assumed to have had unrecorded (and possibly undiagnosed) recurrence before they died [19, 20]. Sensitivity analysis (ii) was fitted using the *msm* package in R, with piecewise constant baseline hazards: the baseline hazard was held constant within the first year and after the first year, but could differ between these two periods [21]. (see Supplement for further details)

## Results

### Description of patient population

Between July 2014 and September 2020, 52,283 eligible patients aged 18 to 98 years, underwent curative major resection. (Supplement Fig. SF1) Of these, 1,803 (3.4%) either died (n = 1793) or were lost to follow up (n = 10) within 9 months of surgery. The remaining 50,480 (96.6%) were included in the study. Patient and tumour characteristics are summarised in Table 1. Mean (SD) age at diagnosis was 68.8 (11.4) years, with 15,496 (30.7%) aged over 75 years. About one third of patients (31.5%) had one or more comorbidities, and this proportion was higher (41.7%) in people aged > 75 years. Patients were less deprived than the population of England as a whole, with 23.8% living in areas of least deprivation and 14.4% living in the most deprived areas. This was more evident in patients aged >75, with 26.4% of older patients living in the least deprived areas and 11.7% living in the most deprived areas (Supplement Table ST2).

Tumours for the majority of patients were histological grade 2 (moderately differentiated) (84.6%) and overall cancer stage 2 (44.1%), with 28.0% having spread to one or more nodes (N-stage > 0) (Table 1) [22]. The primary tumour was colonic in 70.3% of patients. Almost all patients recorded White ethnicity (94.4%) and 55.4% were men.

Compared with younger patients, those who were over 75 were more likely to be women, record ethnicity as White, and have tumours in the colon (Supplement Table ST2). There was some evidence that patients aged over 75 were somewhat more likely to have tumour stage T3 or T4 than younger patients (74.3% vs 65.8%) and less likely to have nodal involvement (25.0% vs 29.3%). Despite this, tumour characteristics were only weakly correlated with age-group overall (Kendall’s τ = -0.01 for overall cancer stage, 0.06 for T-stage and -0.06 for N-stage).

### Exploratory analysis of transitions

Of 50,480 patients who were alive and cancer free at 9 months, 9,517 (18.9%) developed recurrence within 5 years of surgery (Fig. 1).

Of 40,963 patients who did not have recurrence during follow up, 641 (1.6%) died of CRC and 1969 (4.8%) died from non-CRC causes before a cancer recurrence was recorded. The remaining 38,353 (93.6%) patients were alive and recurrence-free at the end of their follow up. Of the 9517 patients who had recurrence, 3599 (37.8%) died from CRC, 921 (9.7%) died from non-CRC causes, and 4997 (52.51%) were alive at the end of their follow up. (See Supplement for specific CRC and non-CRC causes of death)

### Multistate model results overall

Using the multistate model, we estimated the average time spent in each state up to 5 years after major resection. Restricted mean survival time (up to 5 years) was 4.65 years, during which individuals were alive and recurrence-free for 4.28 years and alive with recurrence for 0.37 years on average.

**Fig. 2:**
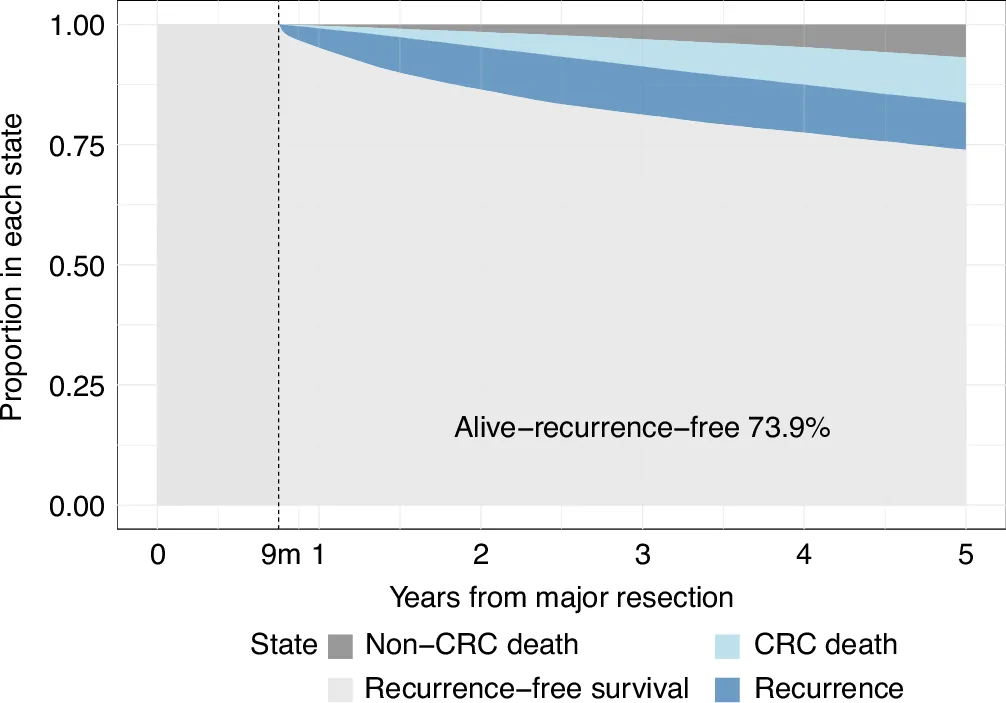
Proportion of patients who survived and were recurrence-free at 9 months after curative resection, and who were in each state over time during the recurrence window (9 months to 5 years after resection). The height of each band represents the proportion who had died from non-CRC causes (dark grey), died from CRC (light blue), were alive with recurrence (dark blue) and alive without recurrence (light grey), over time. Annotated figures are the proportions of patients alive and recurrence-free at the end of the recurrence window (height of the light grey band at 5 years).

Figure 2 shows the proportion of patients in each ‘health’ state over time after curative major resection. Overall, we estimated that 73.9% (95%CI: 73.5%, 74.3%) of patients who had curative major resection were alive and recurrence-free at 5 years after curative major resection (light grey), 9.8% (95%CI: 9.5%, 10.1%) were alive with recurrence (dark blue), 9.4% (95%CI: 9.2%, 9.7%) had died from CRC with or without evidence of recurrence in the data (light blue) and 6.9% (95%CI: 6.8%, 7.1%) had died from non-CRC causes (dark grey). After 1–2 years from curative major resection, the proportion of the full cohort living with recurrence was approximately constant at around 10%.

### Effects of patient and tumour characteristics

#### Overview

**Fig. 3:**
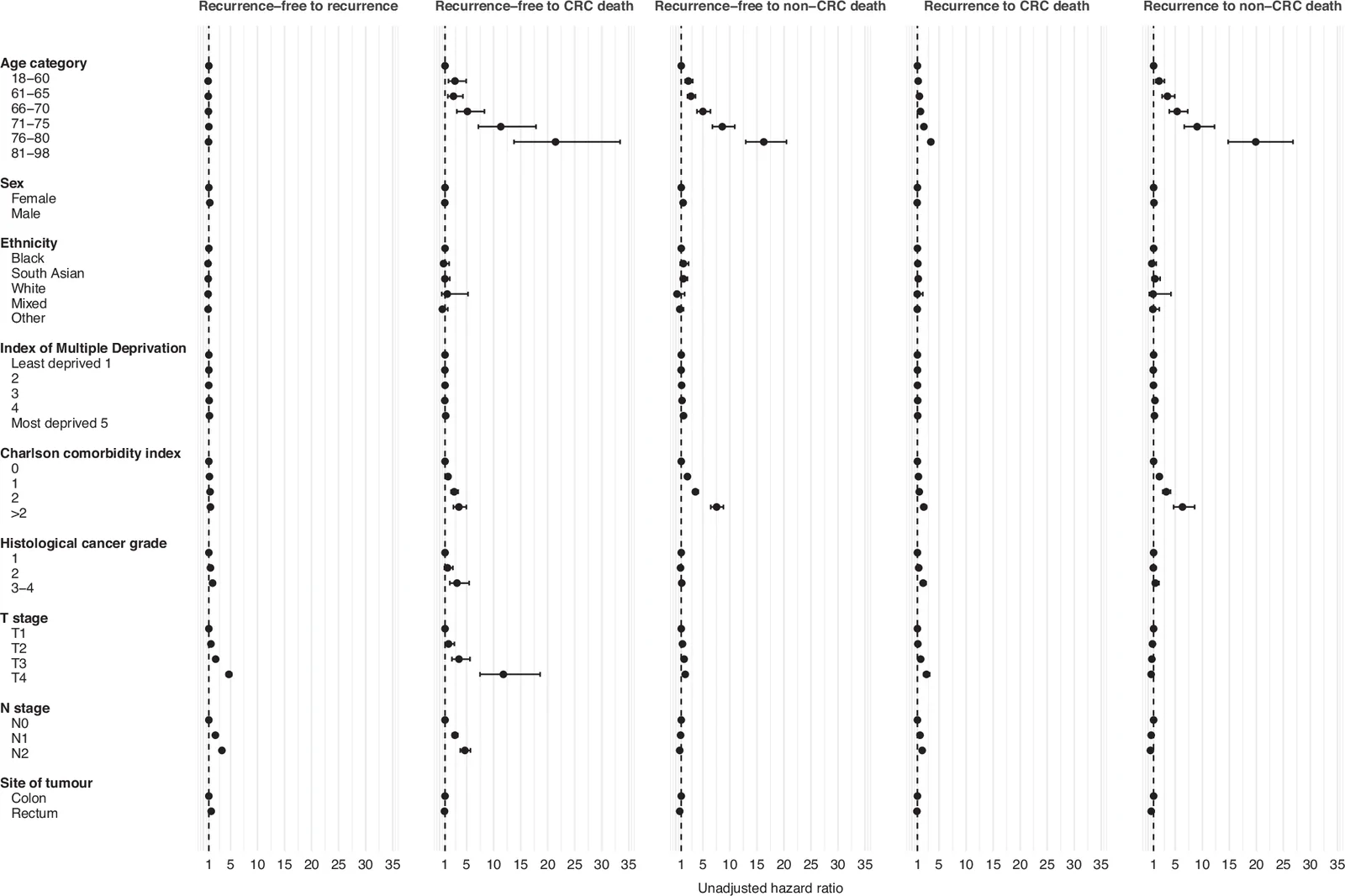
‘Average’ unadjusted hazard ratio estimates (95% confidence intervals) for the five possible transitions in the multistate model: patient characteristics and comorbidities. The first category for each variable is the reference category and has hazard ratio of 1.

Patients aged under 60 and men had only slightly higher recurrence hazard, but there was little evidence that recurrence was associated with ethnicity, IMD or number of comorbidities (Fig. 3). Tumour-related characteristics (higher overall cancer stage, T-stage, N-stage, histological grade) and rectal tumours were the strongest associates of earlier recurrence (Fig. 3).

#### Focus on age

Figure 3 and supplementary Table ST3 show that, although older age at diagnosis was not associated with onset of recurrence, it was strongly associated with both CRC and non-CRC death, whether or not the patient had evidence of recurrence in the data. For example, compared with 18–60-year-olds, patients aged 76–80 had hazard ratio for CRC death after recurrence of 2.19 (95%CI: 1.97, 2.45) and patients over 80 had hazard ratio of 3.48 (95%CI: 3.11, 3.89).

The estimated proportion of patients alive and recurrence-free was 77.4% (95%CI: 77.0%, 77.9%) for the 18-75 age group and 66.2% (95%CI: 65.4%, 67.0%) for the >75 age group. There were 11.3% (95%CI: 10.9%, 11.7%) of younger patients alive with recurrence at 5 years compared with 6.4% (95%CI: 5.9%, 6.8%) of older patients. CRC death occurred in a greater proportion of the older group (13.3% (95%CI: 12.7%, 13.9%) vs 7.7% (95%CI: 7.4%, 8.0%)), due to poorer survival once recurrence was diagnosed. As expected, deaths from non-CRC causes accelerated with age, from 3.6% (95%CI: 3.4%, 3.8%) in the 18–75 age group having this outcome within 5 years of resection, compared to 14.2% (95%CI: 13.6%, 14.8%) in the >75 age group. Patients aged >75 who survived to 9 months after resection, were more likely to die from non-CRC causes than from their cancer by five years after resection (14.2% versus 13.3% respectively).

**Fig. 4:**
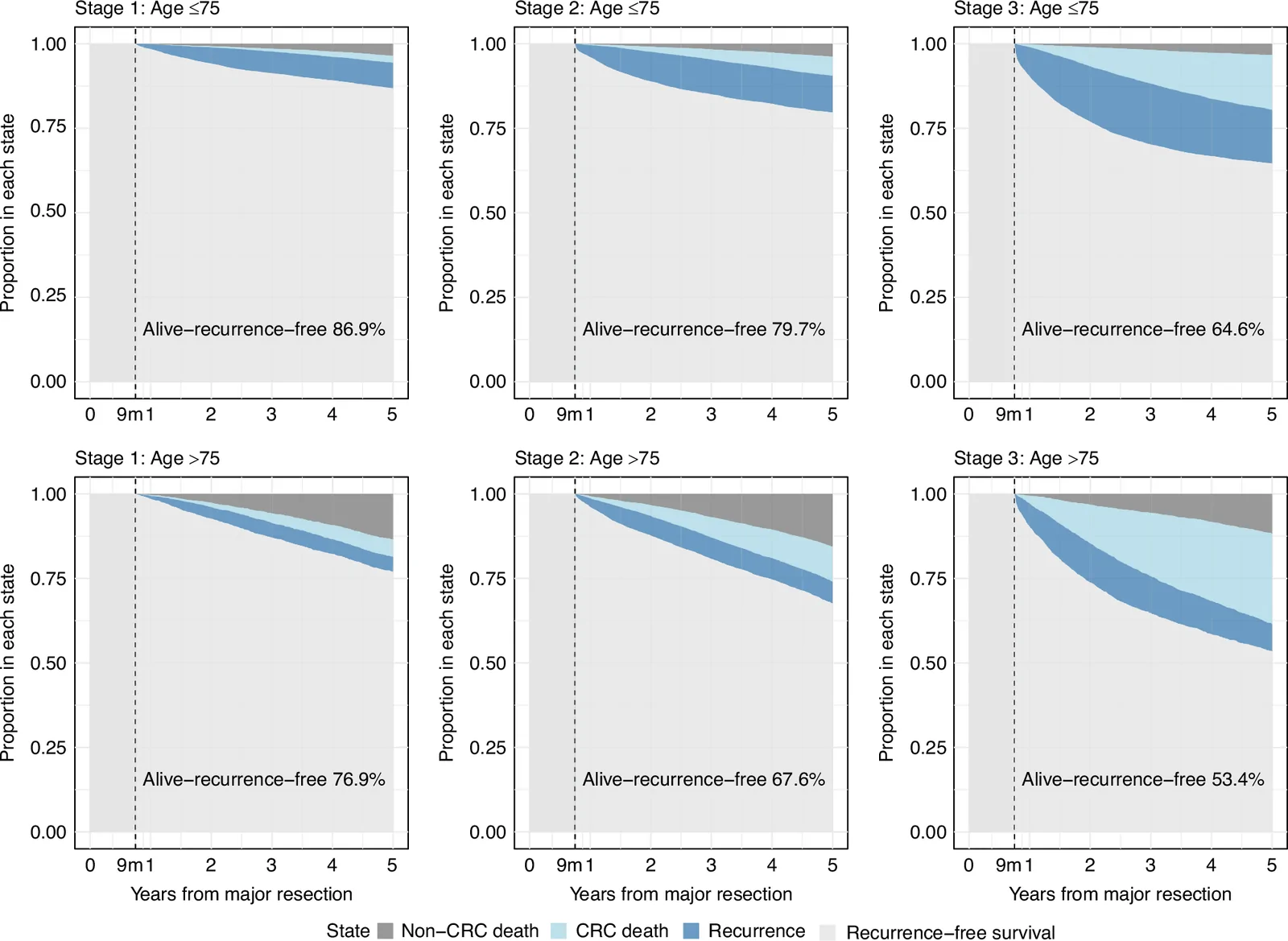
Proportion of patients in each state over time stratified by age group and overall cancer stage. The height of each band represents the proportion who had died from non-CRC causes (dark grey), died from CRC (light blue), were alive with recurrence (dark blue) and alive without recurrence (light grey), over time. Annotated figures are the proportions of patients alive and recurrence-free at the end of the recurrence window (5 years after resection).

Figure 4 shows that overall cancer stage had a larger impact on recurrence-free survival than age. The proportion of patients alive and recurrence-free at 5 years decreased from 86.9% (95%CI: 86.2%, 87.6%) for patients aged 18–75 to 76.9% (95%CI: 75.5%, 78.5%) for patients aged >75, when cancer was detected at an early stage (overall cancer stage 1). In comparison, patients with overall cancer stage 3 had over 20% lower recurrence-free survival in absolute terms, with 64.6% (95%CI: 63.7%, 65.6%) of younger patients and 53.4% (95%CI: 51.8%, 55.2%) of older patients alive and recurrence-free at 5 years.

#### Focus on stage

**Fig. 5:**
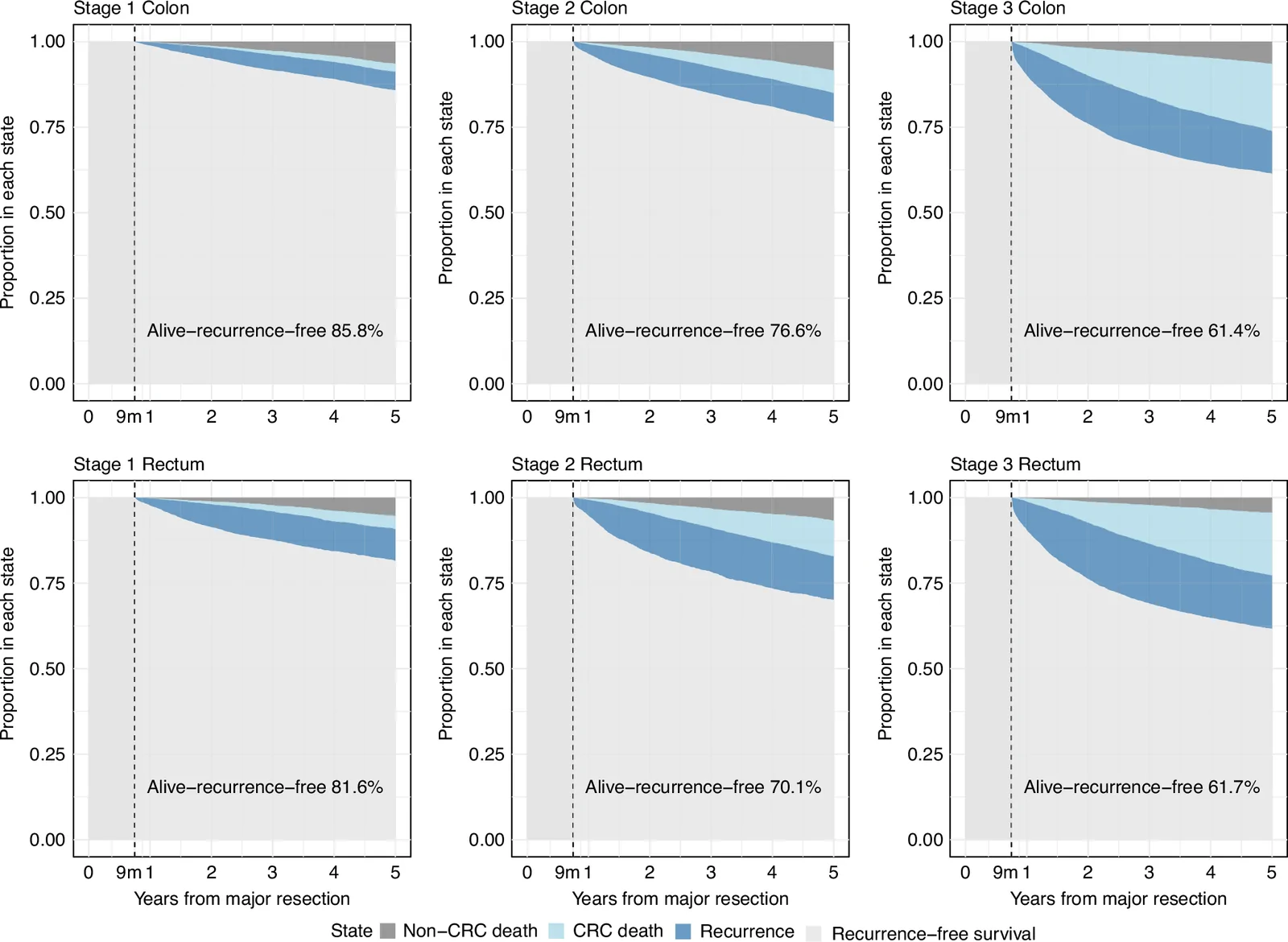
Proportion of patients in each state over time stratified by cancer overall stage and site. The height of each band represents the proportion who had died from non-CRC causes (dark grey), died from CRC (light blue), were alive with recurrence (dark blue) and alive without recurrence (light grey), over time. Annotated figures are the proportions of patients alive and recurrence-free at the end of the recurrence window (5 years after resection).

Figure 3 and supplement Table ST4 show that overall cancer stage, tumour T-stage and N-stage had very large effects on recurrence and CRC death. Figure 5 also shows the overall cancer stage effect is strong for both rectal and colon cancer tumour sites. For overall cancer stage 1, 85.8% (95%CI: 85.0%, 86.6%) and 81.6% (95%CI: 80.4%, 82.7%) of patients with colon and rectal tumours respectively were alive and recurrence-free at 5 years, compared with 76.6% (95%CI: 75.9%, 77.2%) and 70.1% (95%CI: 68.7%, 71.6%) for overall cancer stage 2 tumours, and 61.4% (95%CI: 60.3%, 62.5%) and 61.7% (95%CI: 60.4%, 63.1%) for overall cancer stage 3. Tumour site had less impact on outcomes, with higher risk of recurrence for rectal tumours, but little impact on CRC deaths. The risk of non-CRC deaths was somewhat lower for rectal tumours.

#### Other risk factors

Higher histological grade showed a similar pattern of increasing risk of recurrence and CRC death, but had smaller effects than overall cancer stage. Higher Charlson index was strongly associated with death from both CRC and non-CRC causes, but not with recurrence (Fig. 3). Comorbidities were associated with poorer survival, with just 53.1% (95%CI: 50.0%, 56.4%) of patients with >2 comorbidities alive and recurrence-free 5 years after curative major resection. Figure 3 shows that the hazard ratio due to multi-morbidities increases more steeply for death from non-CRC than for CRC-related deaths. Sex and IMD were not associated with CRC death, but in common with the general UK population, men and people living in more deprived areas had higher risk of death from non-CRC causes. There was no evidence that ethnicity was related to recurrence or survival in this cohort.

### Sensitivity analysis

There were negligible differences in cumulative incidence rates, hazard ratio estimates for baseline characteristics and their 95% confidence intervals after excluding the 641 patients who died from CRC but did not have recurrence recorded prior to death, and when assuming that all CRC-related deaths were preceded by recurrence during life, which may not have been recorded in routine databases. (See supplement for further details).

## Discussion

A new clinical rule-based algorithm to identify recurrence in national health datasets has allowed accurate estimation of 5-year CRC recurrence and mortality in a representative national cohort of patients undergoing curative major resection, presented by age, overall cancer stage and cancer site [4]. Overall, patients who were alive and recurrence-free at 9 months after curative major resection had good prognosis, with 73.9% alive and recurrence-free at 5 years after resection and 9.8% alive with recurrence.

A key finding is that in patients >75 undergoing curative major resection, two-thirds were alive and recurrence-free at 5 years, and patients in this age-group were more likely to die from non-CRC causes than from recurrence by five years (14.2% versus 13.3% respectively). This indicates that other age-related comorbidities are more likely to contribute to risk of death as age at diagnosis increases in this cohort. Patients over 75 make up approximately 30% of those having curative major resection, and this is increasing over time, but they are often excluded from randomised trials and other clinical studies [3, 23]. This study provides evidence for this under-studied group.

As expected, earlier recurrence and death from CRC both before and after recurrence were strongly related to higher overall cancer stage and histological grade [24]. Recurrence-free survival rate at 5 years was 22% higher (absolute difference) for overall cancer stage 3 versus stage 1, which was larger than the difference between patients who were >75 compared to 75 and under (12–13% absolute difference). This suggests that the impact of overall cancer stage is greater in absolute terms than the impact of older age, in this cohort of patients who were selected for curative resection.

Comorbidities more than age were associated with poor survival, with 53.1% of patients with >2 comorbidities alive and recurrence-free 5 years after curative major resection, compared with recurrence-free survival of 66.2% for the >75 age group.

Some results were less expected. For example, it is not clear why higher T-stage should affect non-CRC deaths, or why comorbidities should affect CRC deaths and this may reflect imperfect classification of cause of death. There was little evidence that deprivation was related to recurrence. The absence of an effect of deprivation on CRC outcomes is probably due to the fact that all of these patients were suitable for curative major resection. Within this group there are likely to be fewer social gradients than in the population of all patients diagnosed with CRC. In a review of cancer incidence, Delon et al. showed lower incidence among non-White ethnicities for CRC [25]. However, despite the large sample size overall, due to the age-distribution of the cohorts in our study, the numbers of patients in most ethnic groups were small, which precluded detailed analysis.

### Comparison to other observational studies

Although outcomes after curative major resection for younger CRC patients have been studied extensively, patients over the age of 75 are less well understood. Population studies tend to exclude patients aged 80 or above [3, 23]. Our estimates of recurrence in England were generally consistent with published results from comparable population studies. For patients aged 50–79 years, cumulative incidence of recurrence at 5 years after resection in England was almost identical to the reported cumulative incidence in Denmark (20.5% (95%CI: 20.0%, 20.9%) in England vs 21% (95%CI: 21%, 22%) in Denmark) [3]. There was a larger difference between English and Danish estimates of cumulative incidence at 5 years in patients who had early onset CRC. In England cumulative incidence in patients aged <50 years was 23.5% (95%CI: 21.9%, 25.1%) and in Denmark was 29% (95%CI: 26%, 31%), although both demonstrated higher incidence of recurrence in the early onset group, compared with later onset [3]. Inconsistencies between countries may have arisen due to differences in how recurrence was defined and recorded, or differences in population characteristics. For example, the Danish study defined recurrence from 6 months after resection, with approximately 10% of their cohort having recurrence between 6 and 12 months (see Fig. 2 of Nors et al. [3]). This suggests that some cases of residual primary disease may have been misclassified as recurrence in Denmark, and some very early recurrence may have been missed in England.

Some observational studies have found that early-onset CRC was often diagnosed at a later stage in the disease, when metastases were already apparent [26], resulting in very low recurrence-free survival [27]. This was not a major finding in our study because patients with metastases were excluded, but could explain the slightly higher recurrence in the youngest age group studied (18–60 years, Table 1).

### Comparison to clinical trials

Much of our understanding of outcomes after curative major resection has been informed by randomised clinical trials. However, participants in trials are highly selected, with exclusion criteria related to age, cancer staging, comorbidities and propensity for successful treatment. The MOSAIC and SCOT trials involving Oxaliplatin are good examples. The MOSAIC trial of adjuvant Oxaliplatin recruited 2,246 patients aged 19–75 with overall cancer stage 2–3, and found 3-year recurrence-free survival of 78.2% in the treated group [28]. The SCOT trial randomised 6,088 participants between 20 and 85 years, with overall cancer stage 2–3, to different durations of Oxaliplatin and estimated 3-year recurrence-free survival at 76.7% and 77.1% for the two trial arms [29]. Restricting our analysis to patients with overall cancer stage 2–3, who survived to 9 months (96.5%) and were otherwise unselected, the recurrence-free survival estimate at 3 years was 81.3% (95%CI: 80.7%, 81.9%) for patients aged 18–75 and 75.6% (95%CI: 75.8%, 77.4%) for patients over 75 years. This demonstrates that our estimates are broadly in line with those in clinical trials, although transportation of trial results to clinical practice requires careful attention to patient characteristics as well as tumour staging.

### Strengths

One of the strengths of national health datasets is that they include all patients who have been treated. We quantified the risk of recurrence and death from CRC and other causes at 5 years after resection for a wide range of subgroups, including patients of all ages, sexes, IMD and ethnic groups, and with multiple comorbidities captured by the Charlson Index. Because the times to different outcomes are not independent, we used multistate analysis that properly accounts for competing outcomes. Results were robust to different modelling assumptions about whether or not death from CRC must have been preceded by unrecorded and possibly undiagnosed recurrence before death. Known risk factors for recurrence and cause-specific deaths were confirmed and their relationship with patient and tumour characteristics was quantified. (See supplement for details)

### Limitations

Whilst national health datasets provide opportunities, they lack details of medical history and current care. For example, we did not have information on different follow up and management strategies, detailed pathology or genetic mutations, which limits novel investigation of other important predictors of recurrence and subsequent outcomes. In addition, resection margin status, which is an established prognostic factor in rectal cancer patients undergoing resection, was unavailable in our dataset and could not be evaluated. Although completeness and quality of national health datasets has improved, inaccuracies remain [30].

We used an indicator for cancer recurrence developed by Almilaji et al., which is unlikely to be a perfect measure of incidence and timing of recurrence, and further validation is required. However, the indicator has been validated internally, demonstrating strong association with known recurrence risk factors. Moreover, over 80% of self-reported recurrences from national survey data were captured by the indicator in external validation.

Our study includes only people who underwent surgery. Of eligible patients diagnosed with CRC, 85.1% of those aged ≤75 had surgery, compared with 63.0% of patients aged >75, which is similar to published decreases in selection for curative major resection with age [31]. Older patients who were frail or who had multiple co-morbidities may have been advised against surgery, or may have chosen a less invasive management strategy, so that they do not appear in the analysis. Our results are only generalisable to patients who are considered suitable for curative resection.

## Conclusions

Higher overall cancer stage was the strongest predictor of recurrence and CRC death after curative resection in colorectal cancer patients. Patients aged over 75 make up approximately 30% of those having curative major resection for CRC. Advanced age was not associated with risk of recurrence, but markedly increased the risk of death (from CRC and other causes) following recurrence. At 5 years after curative resection, recurrence-free survival was 66.2% for patients aged over 75, and this group were as likely to die from other causes as from CRC.

## Supplementary information

STROBE checklist

The effect of older age and stage on risk of recurrence and mortality following curative resection, in a national cohort of patients with colorectal cancer Supplement

## Data Availability

Data available from NHS England’s Data Access Request Service (DARS).
