# Supplementary material for: The effect of older age and stage on risk of recurrence and mortality following curative resection, in a national cohort of patients with colorectal cancer
Source: Br J Cancer. 2026 Jul 21;135(8):1267–75. doi: 10.1038/s41416-026-03553-4 (PMC13642942; doi:10.1038/s41416-026-03553-4)
Supplement: Supplementary file 2 [file 41416_2026_3553_MOESM2_ESM.docx]

**The effect of older age and stage on risk of recurrence and mortality following curative resection, in a national cohort of patients with colorectal cancer**

**Supplement**

Table of Contents

[Data sources used in algorithm for incidence and timing of recurrence	2](#_Toc214539742)

[Figure SF1 Flow chart showing creation of the final dataset	3](#_Toc214539743)

[Table ST1: Codes used for recurrence indicator	4](#_Toc214539744)

[Details of analysis of multistate model for recurrence and deaths due to CRC and non-CRC causes	5](#_Toc214539745)

[Observed primary ICD-10 codes for patients who died	7](#_Toc214539746)

[Observed ICD-10 codes defining death from CRC causes	7](#_Toc214539747)

[Observed ICD-10 codes defining death from non-CRC causes	7](#_Toc214539748)

[Table ST2: Comparison of baseline patient and tumour characteristics between age groups	8](#_Toc214539749)

[Figure SF2 Proportion of patients in each state during the recurrence window	9](#_Toc214539750)

[Table ST3: Effects of patient characteristics on transitions: Nelson-Aalen method – multistate model with five transitions	10](#_Toc214539751)

[Table ST4: Effects of tumour characteristics on transitions: Nelson-Aalen method – multistate model with five transitions	11](#_Toc214539752)

## Data sources used in algorithm for incidence and timing of recurrence

1. Cancer Registry (CR). Used to identify all patients newly diagnosed with non-metastatic CRC in England between 1^st^ August 2014 and 30^th^ September 2019. CR included the CRC diagnosis date, patient and tumour characteristics, as well as treatments recorded using the Office of Population Censuses and Surveys Classification of Surgical Operations and Procedures, 4th revision (OPCS-4) codes, and mortality information (linked from Office for National Statistics data) with the last recoded date of death at 22^nd^ June 2022.
2. Hospital Episode Statistics Admitted Patient Care (HES APC). HES APC was linked to CR by patient id to create the cohort and identify episodes of care from two years before to the most available date after the CRC diagnosis. HES APC included diagnosis codes using the international classification of Diseases, 10th revision (ICD-10) codes, and relevant procedures, chemotherapy and radiotherapy treatments using OPCS-4 and ICD-10 codes.
3. Hospital Episode Statistics Outpatients (HES OP): HES OP was linked to the cohort by patient id to capture chemotherapy and radiotherapy treatments recorded using OPCS-4 codes.
4. Systemic Anticancer Therapy (SACT): SACT was linked to the cohort by patient id to identify patients who received cancer chemotherapy in or funded by the NHS in England, in nine months to five years after a curative treatment in the cohort.
5. Radiotherapy (RTDS): RTDS was linked to the cohort by patient id to identify patients who received radiotherapy in nine months to five years after a curative treatment in the cohort.

## Figure SF1 Flow chart showing creation of the final dataset

## Table ST1: Codes used for recurrence indicator

| **Component** | **Database** | **System** |  |
| --- | --- | --- | --- |
| Diagnosis of recurrence | HES-AP | ICD-10 | *Secondary neoplasm:* C77, C78, C79  *Complications of neoplastic disease:* D630, G550, M907, R18, R190, R590 |
| Procedures for local/regional cancer progression | HES-AP | OPCS-4 | *Excision of colon / rectum / anus:* H04, H05, H06, H07, H08, H09, H10, H11, H29, H33, H411, H414, H471, H479, H404,  *Clearance of the pelvis:* X141, X142, X143, X148, X149  *Creation of colostomy/ileostomy:* H151, H152, H158, H159, G742 |
|  | CR |  |  |
| Procedures for metastatic disease | HES-AP | OPCS-4 | *Brain procedures:* A021, A022, A023, A024, A025, A026, A028, A029, A068, A069, A03, A107  *Liver procedures:* J02, J03, J083, J124, J125, J126, J127, J123  *Bone procedures:* V073, V133, W09, W331, W356  *Lung procedures:* E54, E55, E595  *Peritoneum procedures:* T331, T332, T391, T392, T416, T421, T422, T425, T481, T482  *Drainage of peritoneal cavity:* T462  *Omentectomy:* T361 |
|  | CR |  |  |
| Chemotherapy | SACT |  | Any chemotherapy delivery |
|  | HES-AP | OPCS-4 | *Chemotherapy:* X70, X71, X72, X73, X748, X749  *Mode administration of chemotherapy:* X352, X373, X384 |
|  | HES-OP |  |  |
|  | HES-AP | ICD-10 | *Chemotherapy:* Z511, Z512  *Follow-up after chemotherapy:* Z082  *Prophylactic chemotherapy:* Z292  *Recovery after chemotherapy:* Z542 |
|  | HES-OP |  |  |
| Radiotherapy | RTDS |  | Linkage |
|  | HES-AP | OPCS-4 | *Internal radiotherapy of liver:* J123,  *Radiotherapy delivery:* X65  *Preparation for radiotherapy:* X67  *Brachytherapy:* X68, Y354, Y364, Y89  *External beam radiotherapy:* Y91  *Support for radiotherapy:* Y92 |
|  | HES-OP |  |  |
|  | HES-AP | ICD-10 | *Follow-up after radiotherapy:* Z081  *Radiotherapy:* Y842, Z510, Z541 |
|  | HES-OP |  |  |
| Palliative care | HES-AP | ICD-10 | *Palliative care:* Z515 |
|  | HES-OP |  |  |
|  | HES-AP | Administrative  Data* | *Specialty responsible senior clinician palliative medicine: MAINSPEF 315*  *Treatment specialty: RETSPEF 315*  *Destination discharge hospice: DISDEST 88* |

## Details of analysis of multistate model for recurrence and deaths due to CRC and non-CRC causes

In exploratory analysis we plotted the probability of being in each state over time from 9 months to five years separately using the Aalen-Johansen estimate, with all other events treated as censored. These were useful to get a feel for the patterns of movement between states. However, the estimated incidence of events (recurrence, CRC death and non-CRC death) was only correct if competing events could be eradicated, and if competing events were independent, neither of which were true for cancer recurrence and death.

Therefore, we adopted a multistate model to simultaneously estimate the time to recurrence and death from either CRC or other causes (figure 1 in main paper). The model structure included two transient states labelled (1) alive with no recurrence and (2) alive with recurrence, and two absorbing states labelled (3) death from CRC and (4) death from non-CRC cause, from which patients cannot move. There were five possible observed transitions as shown in figure 1.

We assumed that all patients start in state 1, alive without recurrence, at 9 months after resection, because we are interested in patient pathways from the start of follow up. This has been described as the *clock* forward method by Putter et al. We chose the Aalen-Johansen estimate for analysis because it can handle multiple transitions for each patient, entry into subsequent states (including the recurrence state) at any time after the start of follow up and because it does not make parametric assumptions about the baseline hazard. Further, we can implement this method using standard functions in R such as *survfit* and *coxph.* This required first restructuring the data to long format, with one line per transition using the *tmerge* function, and including the patient identifier in the command in order to link each person’s transitions. Once fitted, cumulative incidence of each event, and the probability of being in each state were extracted and plotted against time.

We assumed proportional hazards for the effects of different levels of the baseline characteristics on all transitions. Although proportional hazards are unlikely to be valid for all transitions and for a long follow up period, we considered that the estimated “average” hazard ratios were worthwhile descriptions of the effects of different baseline characteristics.

Model fit was assessed during exploratory analysis by examining plots of Schoenfeld residuals and log-minus-log survival against time (see for example Therneau and Gramsch).

*Sensitivity analysis*

In the primary analysis we modelled observed transitions and assumed that the timing of the observed events was accurate. However, 641 patients died from CRC death, without an indicator of recurrence in the patient records prior to death. This does not seem consistent with the underlying disease process, for which direct transition to death from CRC without first experiencing recurrence is unlikely. This suggests that either these patients were not recurrence-free at the start of follow up, or that recurrence was not recorded and possibly not diagnosed before death. We completed two sensitivity analyses. First, we assumed that these patients were not cancer-free at the start of the recurrence window (9 months after resection) and were excluded from the analysis. Second, we fitted a model that allowed interval censoring, so that any patient who was observed to make the direct transition between recurrence-free and CRC-death was assumed to have unrecorded recurrence prior to death. Models that include interval censoring are more limited because, for some people, we do not know the exact date when recurrence developed. Thus, we assumed that the patient pathway could be modelled as a continuous time Markov process with four health states and four possible transitions as shown in the figure below. As in the original model in figure 1, the four states were: alive without recurrence, recurrence, died from CRC causes, and died from other causes. We assumed that baseline hazards were constant within one year of the start of follow up, and constant from one year from the start of follow up to end of study, but different between these two periods. We assumed proportional hazards for the effects of different levels of the baseline characteristics on all transitions and across the two time periods (0-1 year and >1 year). The Markov models were fitted using the R package *msm*.

*Reference*

Putter H., Fiocco M., and Geskus R. B. 2007. Tutorial in Biostatistics: Competing Risk and Multi-State Models. Stat. In Medicine 26: 2389–2430.

Therneau T. M. and Grambsch **P. M.,*Modeling Survival Data: Extending the Cox Model*. 2000. Springer.**

## Observed primary ICD-10 codes for patients who died

To be consistent with the Office for National Statistics definition of reason for death, we used data from the Hospital Episode Statistics database field called *DEATHCAUSECODE_UNDERLYING*, which records the specific ICD-10 code assigned to the disease or injury that initiated the chain of events leading to death.

### Observed ICD-10 codes defining death from CRC causes

C18: Malignant neoplasm of colon

C19: Malignant neoplasm of rectosigmoid junction

C20: Malignant neoplasm of rectum

C26: Malignant neoplasm of other and ill-defined digestive organs

C26.0: Intestinal tract, part unspecified (Intestine NOS )

C26.9: Ill-defined sites within the digestive system (Alimentary canal or tract NOS , Gastrointestinal tract NOS )

C76: Malignant neoplasm of other and ill-defined sites

C76.2: Abdomen

C76.8: Overlapping lesion of other and ill-defined sites

C77: Secondary and unspecified malignant neoplasm of lymph nodes

C78: Secondary malignant neoplasm of respiratory and digestive organs

C79: Secondary malignant neoplasm of other and unspecified sites(bone, brain, etc)

C80: Malignant neoplasm, without specification of site

C97: Malignant neoplasms of independent (primary) multiple sites

D37: Neoplasm of uncertain or unknown behaviour of oral cavity and digestive organs:

D37.4: Colon

D37.5: Rectum & Rectosigmoid junction

D37.7: Other digestive organs (Anal, Anus NOS, Intestine NOS, Oesophagus , Pancreas)

### Observed ICD-10 codes defining death from non-CRC causes

Patients were considered to have died from non-CRC causes if they were recorded as deceased by the ONS and had none of the above codes in the underlying cause of death.

## Table ST2: Comparison of baseline patient and tumour characteristics between age groups

**Patient and tumour characteristics at diagnosis of CRC for patients aged 18-75 years compared with patients aged greater than 75 years.**

| Variable at baseline | Level | Age 18-75 years  (N=34,984) | Age >75 years  (N=15,496) |
| --- | --- | --- | --- |
| Sex | Male  Female | 20,328 (58.1%)  14,656 (41.9%) | 7,621 (49.2%)  7,875 (50.8%) |
| Ethnicity | Black  South Asian  White  Mixed  Other  Missing | 517 (1.6%)  937 (2.8%)  31,008 (93.4%)  134 (0.4%)  592 (1.8%)  1,796 | 166 (1.1%)  203 (1.4%)  14,469 (96.5%)  27 (0.2%)  128 (0.9%)  503 |
| Index of Multiple Deprivation^1^ | Least deprived 1  2  3  4  Most deprived 5 | 7,909 (22.6%)  7,983 (22.8%)  7,352 (21.0%)  6,303 (18.0%)  5,437 (15.5%) | 4,084 (26.4%)  3,812 (24.6%)  3,288 (21.2%)  2,506 (16.2%)  1,805 (11.7%) |
| Charlson Comorbidity Index^2^ | 0  1  2  >2 | 25,551 (73.0%)  7,103 (20.3%)  1,759 (5.0%)  571 (1.6%) | 9,032 (58.3%)  4,231 (27.3%)  1,587 (10.2%)  646 (4.2%) |
| Histological Grade of cancer | 1  2  3-4  Missing | 1,311 (3.9%)  28,527 (85.7%)  3,436 (10.1%)  1,710 | 532 (3.6%)  12,159 (82.1%)  2,117 (14.3%)  688 |
| Stage of cancer | 1  2  3  Missing | 10,409 (29.8%)  14,217 (40.8%)  10,264 (29.4%)  94 | 3,554 (23.0%)  8,004 (51.8%)  3,900 (25.2%)  38 |
| T stage | T1  T2  T3  T4  Missing | 4,401 (12.7%)  7,484 (21.5%)  18,559 (53.4%)  4,311 (12.4%)  229 | 1,136 (7.4%)  2,823 (18.3%)  8,995 (58.4%)  2,440 (15.9%)  102 |
| N stage | N0  N1  N2  Missing | 24,555 (70.7%)  6,879 (19.8%)  3,298 (9.5%)  252 | 11,551 (75.0%)  2,801 (18.2%)  1,051 (6.8%)  93 |
| Site of tumour | Colon  Rectum | 23,159 (66.2%)  11,825 (33.8%) | 12,347 (79.7%)  3,149 (20.3%) |

All comparisons between the two age groups are statistically significant (p<0.0001) based on Pearson’s test for contingency tables.

^1^UK government’s official measure of relative depravation for small areas in England, divided into quintiles by deprivation index. [<https://assets.publishing.service.gov.uk/media/5a7f0e5ded915d74e33f410b/English_Index_of_Multiple_Deprivation_2015_-_Guidance.pdf> last downloaded 8th October 2025]

^2^Charlson Comorbidity Index, based on a weighted sum of 17 medical conditions.[Charlson ME, Pompei P, Ales KL, MacKenzie CR. *A new method of classification of prognostic comorbidity for longitudinal studies: development and validation.* Journal of Chronic Diseases. 1987;40(5):373–383.]

## Figure SF2 Proportion of patients in each state during the recurrence window

**Observed proportion in each state over time during the recurrence window (9 months to 5 years after resection).**

## Table ST3: Effects of patient characteristics on transitions: Nelson-Aalen method – multistate model with five transitions

| Variable at baseline | Level | Recurrence-free to recurrence HR  1:2 | Recurrence-free to CRC death HR 1:3 | Recurrence-free to non-CRC death HR 1:4 | Recurrence to CRC death HR  2:3 | Recurrence to non-CRC death HR  2:4 |
| --- | --- | --- | --- | --- | --- | --- |
| Age category | 18-60  61-65  66-70  71-75  76-80  81-98 | ref  0.87 (0.81, 0.94)  0.89 (0.84, 0.95)  0.92 (0.87, 0.98)  1.00 (0.94, 1.07)  0.94 (0.88, 1.00) | ref  2.84 (1.64, 4.93)  2.56 (1.52, 4.32)  5.14 (3.18, 8.30)  11.31 (7.18 17.84)  21.44 (13.77, 33.37) | ref  2.32 (1.73, 3.12)  2.80 (2.15, 3.64)  5.01 (3.91, 6.41)  8.59 (6.77, 10.90)  16.27 (12.94, 20.46) | ref  1.14 (1.00, 1.29)  1.36 (1.21, 1.52)  1.56 (1.39, 1.74)  2.19 (1.97, 2.45)  3.48 (3.11, 3.89) | ref  2.02 (1.37, 3.00)  3.55 (2.56, 4.92)  5.35 (3.91, 7.31)  9.04 (6.66 12.25)  19.89 (14.77 26.79) |
| Sex | Male  Female | 1.18 (1.13, 1.23)  ref | 0.96 (0.83, 1.13)  ref | 1.35 (1.23, 1.48)  ref | 0.96 (0.90, 1.03)  ref | 1.06 (0.92, 1.21)  ref |
| Ethnicity | Black  South Asian  White  Mixed  Other | ref  0.85 (0.68, 1.05)  0.88 (0.74, 1.03)  0.86 (0.58, 1.27)  0.86 (0.68, 1.09) | ref  0.73 (0.30, 1.76)  0.98 (0.51, 1.89)  1.41 (0.38, 5.24)  0.51 (0.17, 1.52) | ref  1.39 (0.82, 2.35)  1.38 (0.89, 2.14)  0.22 (0.03, 1.62)  0.73 (0.38, 1.42) | ref  1.07 (0.75, 1.51)  1.15 (0.88, 1.49)  0.98 (0.48, 2.00)  0.97 (0.65, 1.43) | ref  0.66 (0.29, 1.47)  1.25 (0.71, 2.21)  0.90 (0.19, 4.20)  0.87 (0.37, 2.05) |
| Index of Multiple Deprivation | Least dep 1  2  3  4  Most dep 5 | ref  1.00 (0.95, 1.07)  0.98 (0.92, 1.04)  1.06 (1.00, 1.13)  1.11 (1.04, 1.19) | ref  0.97 (0.78, 1.22)  1.00 (0.79, 1.26)  0.96 (0.75, 1.23)  1.12 (0.87, 1.45) | ref  0.98 (0.86, 1.12)  1.07 (0.94, 1.23)  1.15 (1.00, 1.32)  1.42 (1.23, 1.63) | ref  1.01 (0.91, 1.11)  1.01 (0.91, 1.12)  1.05 (0.94, 1.16)  1.05 (0.94, 1.17) | ref  0.93 (0.76, 1.15)  0.97 (0.79, 1.20)  1.25 (1.02, 1.54)  1.15 (0.92, 1.43) |
| Charlson index | 0  1  2  >2 | ref  1.11 (1.06, 1.17)  1.23 (1.14, 1.33)  1.29 (1.14, 1.45) | ref  1.56 (1.30, 1.87)  2.70 (2.13, 3.43)  3.56 (2.55, 4.95) | ref  2.14 (1.93, 2.37)  3.62 (3.17, 4.14)  7.55 (6.47, 8.81) | ref  1.19 (1.09, 1.29)  1.34 (1.17, 1.53)  2.17 (1.78, 2.66) | ref  2.06 (1.76, 2.41)  3.34 (2.70, 4.13)  6.34 (4.69, 8.57) |

## Table ST4: Effects of tumour characteristics on transitions: Nelson-Aalen method – multistate model with five transitions

| Variable at baseline | Level | Recurrence-free to recurrence HR  1:2 | Recurrence-free to CRC death HR 1:3 | Recurrence-free to non-CRC death HR 1:4 | Recurrence to CRC death HR  2:3 | Recurrence to non-CRC death HR  2:4 |
| --- | --- | --- | --- | --- | --- | --- |
| Histological Cancer grade | 1  2  3-4 | ref  1.30 (1.15, 1.47)  1.69 (1.48, 1.93) | ref  1.46 (0.88, 2.45)  3.21 (1.88, 5.47) | ref  0.87 (0.70, 1.08)  1.09 (0.86, 1.40) | ref  1.24 (1.00, 1.54)  2.05 (1.62, 2.58) | ref  0.95 (0.65, 1.39)  1.31 (0.87, 1.99) |
| Stage of cancer | 1  2  3 | ref  1.74 (1.64, 1.85)  3.70 (3.48, 3.92) | ref  2.62 (1.98, 3.47)  6.59 (5.02, 8.65) | ref  1.43 (1.29, 1.59)  1.06 (0.93, 1.20) | ref  1.62 (1.42, 1.85)  2.37 (2.08, 2.69) | ref  0.86 (0.72, 1.02)  0.45 (0.37, 0.54) |
| T stage | T1  T2  T3  T4 | ref  1.39 (1.26, 1.54)  2.26 (2.06, 2.47)  4.71 (4.28, 5.18) | ref  1.65 (0.99, 2.74)  3.57 (2.27, 5.61)  11.79 (7.47, 18.60) | ref  1.23 (1.02, 1.47)  1.53 (1.30, 1.80)  1.74 (1.44, 2.12) | ref  1.08 (0.86, 1.35)  1.60 (1.32, 1.95)  2.67 (2.19, 3.26) | ref  0.78 (0.58, 1.04)  0.67 (0.52, 0.87)  0.54 (0.40, 0.72) |
| N stage | N0  N1  N2 | ref  2.21 (2.11, 2.32)  3.42 (3.23, 3.62) | ref  2.83 (2.37, 3.38)  4.67 (3.81, 5.72) | ref  0.88 (0.78, 1.00)  0.72 (0.58, 0.88) | ref  1.48 (1.37, 1.60)  1.88 (1.73, 2.06) | ref  0.55 (0.47, 0.66)  0.41 (0.32, 0.52) |
| Site of tumour | Colon  Rectum | ref  1.44 (1.38, 1.50) | ref  0.90 (0.76, 1.07) | ref  0.72 (0.65, 0.80) | ref  0.91 (0.85, 0.98) | ref  0.56 (0.48, 0.65) |
